# Localization of health systems in low- and middle-income countries in response to long-term increases in energy prices

**DOI:** 10.1186/1744-8603-9-56

**Published:** 2013-11-07

**Authors:** Sarah L Dalglish, Melissa N Poulsen, Peter J Winch

**Affiliations:** 1 Social and Behavioral Interventions Program, Department of International Health, Johns Hopkins Bloomberg School of International Health, 615 N. Wolfe St., Baltimore, MD 21205, USA

**Keywords:** Health systems reform, Climate change, Decentralization, Rural health, Localization, Access to care, Developing countries

## Abstract

External challenges to health systems, such as those caused by global economic, social and environmental changes, have received little attention in recent debates on health systems’ performance in low-and middle-income countries (LMICs). One such challenge in coming years will be increasing prices for petroleum-based products as production from conventional petroleum reserves peaks and demand steadily increases in rapidly-growing LMICs. Health systems are significant consumers of fossil fuels in the form of petroleum-based medical supplies; transportation of goods, personnel and patients; and fuel for lighting, heating, cooling and medical equipment. Long-term increases in petroleum prices in the global market will have potentially devastating effects on health sectors in LMICs who already struggle to deliver services to remote parts of their catchment areas. We propose the concept of “localization,” originating in the environmental sustainability literature, as one element of response to these challenges. Localization assigns people at the local level a greater role in the production of goods and services, thereby decreasing reliance on fossil fuels and other external inputs. Effective localization will require changes to governance structures within the health sector in LMICs, empowering local communities to participate in their own health in ways that have remained elusive since this goal was first put forth in the Alma-Ata Declaration on Primary Health Care in 1978. Experiences with decentralization policies in the decades following Alma-Ata offer lessons on defining roles and responsibilities, building capacity at the local level, and designing appropriate policies to target inequities, all of which can guide health systems to adapt to a changing environmental and energy landscape.

## Introduction

Discussions on strengthening health systems in low- and middle-income countries (LMICs) have typically focused on overcoming internal challenges such as program integration, financial sustainability, quality of care, and shortages of trained health care workers [1-8]. These issues warrant attention; however, the current debate largely bypasses external threats emanating from global economic, social and environmental changes. In recent years, public health experts have highlighted the need to adapt health systems to manage the adverse outcomes of climate change [9] and reduce greenhouse gas emissions [10], but the health literature has only begun to grapple with the depletion of conventional petroleum reserves and the rising prices and energy insecurity that will occur during any transition to a new energy regime [11].

The availability of plentiful and cheap energy in the form of fossil fuels such as petroleum is thought to have significantly contributed to many health gains made over the past century [11,12]. However, these advances are likely to be threatened in coming decades as conventional petroleum reserves reach peak production – an event which the International Energy Agency (IEA) estimates will occur as early as 2020, resulting in rising energy prices [13,14]. Rising prices for petroleum resources will affect health systems through the use of petroleum-based commodities, increased transportation costs, and effects on health facility operations, among other pathways [11,15,16]. Current health systems are not prepared to respond to this global environmental threat, particularly in resource-poor settings in LMICs.

We propose the localization of health systems in LMICs as one facet of the health sector’s response to rising petroleum prices and resulting energy insecurity. “Localization” refers to the production of the basic inputs of health – including energy, commodities, and human resources – at local and regional scales whenever possible, preserving scarce energy resources for inputs whose production cannot realistically be localized. Localization also contributes to mitigating global climate change by reducing carbon emissions created by burning fossil fuels. There are multiple intersecting justifications for localization policies, including preservation of biodiversity, promotion of sustainable food systems, and climate change mitigation. In this article we focus on how localization helps maintain health system functionality in the face of long-term trends of increasing prices for fossil fuels, as this topic has rarely been addressed in the literature [11].

By following principles of localization, health systems can increase their resilience to external threats such as impending resource constraints and energy insecurity while promoting values that have guided debates over primary health care since the 1978 Alma-Ata Declaration on Primary Health Care, such as equitable access, community participation, and sustainability. Though the decentralization policies put in place in the decades following Alma-Ata focused on a transfer of competencies and decision-making power from central to more peripheral levels, rather than the localized production of health inputs and services, they can offer valuable lessons about organizing and providing health care at lower levels of the health system.

## Review

### Health systems in the face of rising petroleum prices

The timing of “peak oil” is the subject of debate within the scientific and policy literatures; however, it has become clear that peak discovery of petroleum occurred approximately 40 years ago, and most estimates locate peak production within the first half of this century [11,12,17]. The International Energy Agency (IEA) recently predicted a peak in 2020 [13,14]. As deposits of easy-to-extract oil (conventional oil) are exhausted, there is an ongoing transition toward exploiting unconventional fossil fuel reserves in the form of oil sands, oil shale, offshore oil and hydraulic fracturing for gas. Reserves of unconventional oil are estimated by the IEA to be large, and with reserves of this size we might expect prices to drop considerably [13,14,18]. However, extraction and refining of oil from these unconventional sources is more difficult – and thus more expensive – contributing to higher prices [13,14,18,19].

Concurrently, global dependency on fossil fuels is increasing due to a combination of population growth and economic growth in LMICs. Not only is demand for energy resources growing fastest in LMICs and rapidly emerging markets, this is also where most population growth will occur in coming decades [20]. The IEA has made varying predictions of future growth in energy demand between 2010 and 2030, but all include significant increases led by increased demand in rapidly growing middle income countries such as China and India [13,14]. Prices will rise as demand outpaces supply [17,21], putting in jeopardy both the functionality of health systems and the livelihoods of the populations they serve.

These circumstances will disproportionately affect poor areas in LMICs, where energy insecurity is already a major burden on health systems and energy poverty, or lack of reliable access to modern forms of energy, affects billions of people [22]. While new petroleum and other energy resources will undoubtedly be developed, the rise in prices as conventional reserves are exhausted will price disadvantaged buyers out of the market – just as famines can occur among disenfranchised populations in the context of ample food supplies [23]. Thus, health systems must adapt to the long-term rise in fossil fuel prices with two considerations in mind: 1) the systems’ operations and organization, and 2) how households and populations will be affected by energy insecurity in terms of evolving health needs and difficulty accessing services. In this article we focus mainly on the former; however, we also acknowledge the effects on populations’ livelihoods and modes of accessing health care since these will determine how health systems reach their intended beneficiaries.

### Effects on health sector operations and organization

Contemporary health systems are dependent on affordable petroleum resources for the maintenance of supply chains, supervision systems and referral of patients to higher levels of care [11,16]. Energy is also needed for basic operations such as refrigeration, lighting and medical equipment, yet supply is often limited in lower-level health facilities in the developing world. Worldwide, it is estimated that 1 billion people are served by health facilities without electricity [24]; for example, in Uganda, only 1 percent of rural outpatient clinics are connected to the electric grid [25]. Furthermore, even where access to electricity exists it is often unreliable: in a systematic review of African health facilities’ access to electricity, only 28% of facilities with electricity reported a reliable supply and in some countries such as The Gambia, up to a third of facilities rely on generators [26]. Spoilage of vaccines due to flaws in the cold chain is widely considered to be a major problem, and a key limitation to full implementation of the Global Immunization Vision and Strategy (GIVS) [27]. Facilities without electricity often rely on paraffin lamps or candles that offer low-quality light, rendering medical procedures dangerous after nightfall [25,26]. Health workers are also scarce in rural environments: remoteness and difficulty of access tend to be strongly correlated with absenteeism among health personnel [28].

A 2011 special issue of the *American Journal of Public Health* (AJPH) on Peak Petroleum and Public Health highlighted the myriad ways that increasing energy insecurity will impact public health [15,19], and other authors have recently analyzed the effects of peak oil on health and health systems in LMICs [11,16]. They describe several pathways by which rising petroleum prices will affect health in LMICs, including infrastructure and supplies, transportation and referral systems, energy for health services, human resources policy and health systems organization (Table 1). Seemingly fundamental aspects of operations will likely be challenged: Frumkin points out that modern antiseptic practice is based on the use of disposable materials (e.g. syringes, intravenous solution bags, rubbing alcohol, sterile wrapping), which are petroleum-based [15]. Shortages of medical goods have already been observed during historical periods of petroleum scarcity, such as shortages of syringes during the 1973 oil crisis and even rising prices of Band-Aids [29]. While data on supply procurement in hospitals in low-income countries is scarce, low-income countries generally import medical supplies and will thus be particularly vulnerable to supply shortages and price hikes [16].

**Table 1:** Effect of rising petroleum prices on the functioning of health systems in LMICs

	***Effects of rising petroleum prices***
*1. Health infrastructure, supplies, & equipment*	• Increased cost of petroleum-based medical supplies and equipment (e.g. rubber gloves, syringes, pharmaceuticals)
	• Increased cost of transporting construction materials, equipment, and other commodities to remote parts of the health system
*2. Transportation and referral system*	• Disruptions to medical supply chains
	• Increased fuel costs for transporting health workers and administrators
	• Challenges to referral systems as patient transportation becomes more costly
*3. Energy for health services*	• Disruption to fossil fuel-dependent health facility operations (e.g. heating/cooling, powering medical equipment, lighting)
*4. Human resources policy*	• Personnel shortages in rural areas and increased absenteeism
	• Increased costs of supervisory visits to remote areas
*5. Health systems organization*	• Centralized health systems increasingly inaccessible to rural or remote populations
	• Less effective administrative and personnel supervision in peripheral areas

Rising petroleum prices may also threaten the current structure and organization of health systems, which rely on the ready transport of drugs and essential commodities, as well as people, through referral and supervision systems. Already fragile supply chains and referral systems will be weakened [16] and increased transportation costs will exacerbate the inequitable distribution of human resources, reinforcing oversupply in cities at the expense of rural areas. Indeed, health systems in rural areas will be disproportionately affected by increasingly burdensome financial costs of patient and staff transportation. Centralized systems requiring frequent transport of goods and people will become less sustainable, requiring a re-thinking of the optimal organization of the health system overall. Such changes will be particularly onerous for patients suffering from diseases requiring specialized care that is often centralized in large urban centers, such as cancer, or that call for frequent clinical encounters, such as many types of mental illness. The fact that rising rates of non-communicable disease and mental illness in LMICs will coincide with rising petroleum prices will pose a major challenge in the coming years and decades.

### Household energy insecurity and access to health services

Poor populations in LMICs will be most affected by rising energy prices following the depletion of conventional petroleum reserves, as they lack resources to adapt to drastic social and economic changes. The poor, ethnic minorities and other disadvantaged populations are already sensitive to minor fluctuations in the prices of fuel, food, and other essential goods; they are also more likely to suffer from environmental exposures (weather events, natural disasters, etc.) and be denied access to health care [11,30]. Increasing energy prices will cause harm to rural livelihoods, for example via the limited availability of inputs derived from fossil fuels such as fertilizer and the increased cost of transporting produce [31]. The main effect of the changing energy environment on the health of populations may be via worldwide slowdowns in economic growth and employment [12]; others have warned of growing food insecurity as globalized food production systems are disrupted by rising input and transport costs [32]. Rising petroleum prices will also exacerbate existing inequalities between nations, with landlocked developing countries suffering from even greater barriers to economic growth in the form of higher transport costs [33]. Low-density landlocked countries like those of the Sahel (Mali, Niger, Chad) will be especially hard-hit and rural populations risk becoming more isolated than they are currently [33].

As within health systems, rising petroleum prices will affect households’ transportation options, with potentially dire effects for access to health care. In sub-Saharan Africa, only 30% of the rural population currently has access to adequate transport [34] and rural transport charges are higher than in any other region of the world [35]. Many rural populations live in a “walking world,” and the burdens imposed by inadequate and unreliable transportation fall particularly hard on women [35]. Furthermore, medical referral systems in rural areas are often barely functional, with significant mortality resulting from failed referrals [36]. Some authors have operationalized the effects of rising petroleum prices on transportation-related barriers to accessing health care using the “three delays” model, showing that lack of transportation is a cause of many maternal deaths [16,28].

## Localization

While there have been a number of review articles on the consequences of dwindling conventional petroleum reserves on public health and the health sector [11,12,15,16,28], there have been fewer attempts to map solutions and describe how health systems should adapt to changing energy availability and prices. It is clear that many different types of solutions will be required, across all sectors. Within the health sector, localization will be an important part of the response. By complementing efforts to decentralize healthcare and reinforce peripheral health service delivery, localization can help health systems adapt to projected long-term trends of increasing cost of fossil fuels.

### Defining localization

“Localization” refers to a system in which the production of basic inputs for survival such as food, water, and energy production occurs locally or regionally rather than at a centralized site [37]. Frequently applied to food and energy production, the concept of localization stems from the idea of “bioregionalism” originally introduced by Berg and Dasmann in the 1970s [38]. They conceptualized a bioregion as an area defined by a unique pattern of natural characteristics (e.g. climate, landforms, watersheds, soils, native plants and animals) and included people as an integral aspect of a place [39]. They proposed the need to “live in place” within one’s bioregion by “following the necessities and pleasures of life as they are uniquely presented by a particular site, and evolving ways to ensure long-term occupancy of that site” [38]. In the environmental sustainability literature, localization is framed as building community resilience to peak petroleum and mitigating climate change by 1) increasing local production of goods and services, and 2) decreasing reliance on inputs from outside the local area.

In the context of health systems, localization involves moving the production of goods and services used in the health sector from global to more local scales.

#### Definition of localization of health systems

Localization is a set of processes moving production of health and input goods for health (goods, services, human resources), as well as responsibility and oversight for these processes, to more local or regional scales.

Health services are not only adapted to local needs, but also to local environmental conditions and resource availability, with much less reliance on petroleum-based and globalized resources. Furthermore, local workers and citizens participate in the “co-production” of the health system, a term from the development literature that describes situations when “citizens … play an active role in producing public goods and services of consequence to them” [40]. Collaboration between government and civil society can be particularly important in contexts of weak state authority and public service delivery – the same conditions that contribute to low levels of electrification and other forms of energy insecurity [41].

### What would localization of health systems look like?

Localized health systems aim to provide essential health care services to populations where they live, in a manner that is sustainable given rising prices for petroleum products and energy. Localization is a framework for health services to be produced and provided locally, including at the most peripheral levels, drawing on local resources as much as possible. By incorporating elements of localization, health systems can increase their resilience to external threats such as those posed by impending resource constraints. In Table 1 above, we outlined how rising petroleum prices and energy insecurity will affect five aspects of health systems: health infrastructure, supplies and equipment; transportation and referral; energy for health services; human resources; and health systems organization. In this section we offer a framework for responding to these challenges, give examples of localized solutions in LMICs and suggest ways to ensure steady supplies of the inputs to health given rising petroleum prices (summarized in Table 2).

**Table 2:** Localized solutions to rising petroleum prices in the health sector in LMICs

	***Examples of localized solutions to rising petroleum prices***
*1. Health infrastructure, supplies, & equipment*	• Build infrastructure using locally available materials
	• Reduce reliance on disposable materials and move toward sterilizing reusable ones on-site
	• Substitute local goods when possible, including those made from traditional materials (bandages, etc.) of known safety and efficacy
*2. Transportation and referral system*	• Create a strategic fuel reserve to power emergency vehicles
	• Identify alternative means of transport for referral systems (local ambulance schemes, animal-powered referrals, etc.)
	• Use distance technology e.g. cell phone-based monitoring systems when feasible and appropriate
*3. Energy for health services*	• Identify and utilize local energy resources (solar roof panels, hydropower, wind power)
	• Utilize architectural design allowing for natural lighting, heating & cooling
	• Adopt energy efficient technologies & reserve fuel supplies to ensure supply chains for goods whose production cannot be localized
*4. Human resources policy*	• Scale up task-shifting to CHWs and increase roles for local health workers
	• Provide incentives for health workers to stay in rural communities
	• Scale-up training activities and training of trainers at the lowest levels of the health system
	• Localize supervisory structures and/or provide non-petroleum based forms of transportation for supervisory visits
*5. Health systems organization*	• Create satellite health facilities or health posts in rural or peripheral areas
	• Clarify and improve upon decentralization policies using evidence-based practices
	• Empower local governance by creating village health committees, health facility oversight boards, and financing and procurement systems that can support and monitor implementation of health services

Putting localization into practice means that different solutions are necessary in different places. Instead of following a single model, localized health systems will be adapted to fit local ecosystems and meet local needs, with participation by local communities. Regarding *health infrastructure and supplies*, decisions in a localized health system would depend on the area’s resources and climate, for example in terms of utilizing locally-available and sustainable building materials (brick, mud, cement, etc.) and traditional architecture to save on heating/cooling costs. Local production of health system inputs could also include blankets, bandages and other simple products. In most cases pharmaceuticals will need to be supplied regionally or internationally, though the local production of traditional indigenous medicines could potentially play a role. Supplying the health system locally will engender a number of non-health related benefits, such as creating local jobs, contributing to local economies, and building community connections between consumer and producer.

The greatest proportion of petroleum use globally is in *transportation*, and as conventional petroleum reserves are exhausted, the rise in transportation costs will present perhaps the greatest challenge to health systems [12]. Indeed, rising transportation costs may ultimately force the issue of localization by undermining basic functions of the health system. Supply chains in LMICs are already overburdened and unreliable in many places, and given that some supplies and equipment will always need to be transported to more peripheral or remote locations, it will become even more important to reserve petroleum fuel for these essential items [16]. The health system also relies on petroleum to transport personnel and patients. However, even at times when fuel has been relatively affordable, delayed or failed referral has been a cause of many deaths in LMICs [28]. Rising transportation costs will represent an increasingly high barrier to accessing health care among the rural poor in particular. It will thus become important to design sustainable transportation solutions and consider options such as animal-powered transportation, radio-ambulance systems for patients [36], or coordination with other branches of local service delivery. The use of mobile devices such as phones to support medical and public health goals, known as mHealth, will also be an important part of the solution, for example by facilitating surveillance, supply chain management, treatment compliance, and public health awareness, and monitoring quality of care and hospital attendance [42]. These strategies may help to reduce the reliance on transportation of medical personnel, for example for monitoring and evaluation purposes; however, in most cases telemedicine or mHealth technologies are unlikely to replace the clinical encounter.

Even basic health services require reliable energy for lighting, heating/cooling, and medical equipment, and local or on-site *energy production* is an essential feature of localized health systems. Some programmatic approaches to electrification for health services have been proposed, though improved non-petroleum energy sources for health systems remain rare in rural areas [24,43]. Technology has an important role to play in harnessing local energy resources, which could include wind, solar or hydroelectric. Energy production at the local level of health systems has some interesting precedents, notably in the use of biogas systems both to safely process hospital and human waste and generate methane gas for on-site use as fuel [44,45]. In such systems, organic waste and latrine outputs are mixed in an airtight digester, where they are broken down by anaerobic bacteria. A study in India showed that adding blood-contaminated hospital waste (cotton dressings, solid plaster, linens, etc.) increased the quantity and quality of biogas while simultaneously solving the problem of placenta disposal [46]. On-site production of energy is preferable, as energy sources like liquefied petroleum gas (LPG) can be difficult to obtain in rural areas [24,43].

Localized health systems’ *human resources policy* will also need to make use of local resources. A growing body of evidence suggests the efficacy of programs using Community Health Workers (CHWs) for a number of interventions, with significant reductions in child mortality, particularly in areas at the periphery of health systems [47-49]. In principle, CHWs are recruited to work in their own communities, providing an example of localized human resources for the new energy paradigm. Generating links between the health system and the community it serves can have positive impacts for health in rural areas. For example, a project in Uganda using solar-powered radios to link traditional birth attendants with the formal health system to provide advice and referral for difficult deliveries nearly halved maternal mortality in three years [50]. Distance technology or telemedicine may also be an option in some settings [16], though in many cases local human resources and health expertise will be more reliable and cost-effective. Further, given the increasing cost of training and supervisory visits, health workers at the local level will need to have greater responsibility for planning programs and monitoring results. This represents a significant shift; a transition toward such a novel human resources paradigm would require significant capacity building and new institutional, financial and technological frameworks – no small undertaking [15,19]. Quality of care will also be a significant concern, and indeed the existing evidence is somewhat mixed on the quality and effectiveness of lay health workers providing various health interventions, suggesting that systems of monitoring and assuring quality of care will be essential to future permutations in human resources policy [49].

Thus, the challenges created by rising petroleum energy prices imply the need for fundamental changes to *health systems’ organization and structure*. Localization aims to re-shape health systems to bring health to people where they live by creating satellite health facilities in local communities, increasing roles for CHWs and other local staff, and establishing community governance bodies. This expansion of the health system must be accompanied by reforms in governance, with the creation of bodies such as community oversight committees and village health boards that exercise real power in local monitoring, financing and procurement systems [51,52]. Some of these transformations were envisaged at Alma-Ata in 1978, and while policy changes were effected, the top-down structure of health systems was not truly challenged. It remains to be seen whether high energy prices resulting from the depletion of conventional petroleum reserves may succeed where rhetoric failed by forcing health systems to rely upon local resources. Furthermore, processes of localization may also benefit from a renewed emphasis on preventive care, so as to reduce the burden on health systems struggling to face increasing energy prices.

#### Lessons from decentralization policies

Local action to empower local communities became a strong theme of global public health with the 1978 International Conference on Primary Health Care in Alma-Ata, and decentralization was hailed as one pathway to achieve this goal [53]. Decentralization refers to the transfer of fiscal, administrative and political authority for health service delivery from the central levels of the Ministry of Health to alternate institutions, frequently district health systems [54]. Cohen and Peterson summarize decentralization policies as some combination of 1) moving civil servants from central locations to sites closer to the users served or resources administered; 2) increasing the decision-making authority of local level administrators; and 3) increasing the authority of local users and other stakeholders to make decisions [55]. Significant attempts to decentralize public health services began in the 1970s; however, decentralization is not unanimously embraced within the health sector. Critics have argued that the sector is too complicated for local control and that greater local-level autonomy may conflict with the objectives of the overall health system [56]. In this section, we compare the concepts of decentralization and localization and draw lessons from the former to inform implementation of the latter.

### Comparing decentralization and localization

In Table 3, we compare the concepts of decentralization and localization in terms of their definitions, the problems they seek to solve, potential benefits, and published applications to health systems. As discussed above, the overarching aims of both concepts are to create sustainable systems, improve services’ quality and efficiency, increase access to care, promote local participation and decision-making, and render more transparent the mechanisms of service delivery. However, in the case of decentralization, sustainability primarily refers to fiscal sustainability, whereas localization seeks to create health systems based on ecologically sustainable levels of resource consumption and increase the role of local communities in producing the inputs of health. Indeed, localization identifies environmental sustainability as a key concern and promotes energy self-sufficiency alongside the broader administrative self-sufficiency conceived of by decentralization. Furthermore, localization of health systems is a much more recent concept, with no published applications related to health systems in LMICs and only recent work on developed-country health systems [11,57].

**Table 3:** Decentralization and localization as guiding principles for health systems in LMICs

	**Decentralization**	**Localization**
Definition	A set of policies that 1) move civil servants from central locations to sites closer to the users served; 2) increase decision-making authority of local administrators; and 3) increase decision-making authority of local users [55]	A set of processes that move production of health and input goods for health (goods, services, human resources), as well as responsibility and oversight over functioning, to more local or regional scales.
Problems identified	- Failure to adapt interventions to local needs	- Increasing energy prices and petroleum scarcity
	- Low quality of services at the periphery	- Lack of decision-making power at local level
	- Lack of decision-making power at local level	- Need to involve local communities as stewards of local resources
Potential benefits	- Improvements in equitable distribution of health care	- Adaptation to rising energy prices and mitigation of climate change
	- Accountability of decision-making	- Empowerment of local actors
	- Financial sustainability of health systems	- Contribution to local economies
		- Environmental sustainability of health systems
Application to health systems	- World Health Organization [58,59]	- Frumkin et al. 2009, Pubic Health Reports [11]
	- World Bank [60]	- Hess et al. 2011, American Journal of Public Health [57]
	- Bossert and Mitchell 2011, Social Science and Medicine [56]	

Both decentralization and localization prescribe shifting activities to the local scale in recognition that solutions must be tailored to local needs and conditions. However, one key difference between the two lies in the process for achieving the end goal of improved health services for poor or remote populations. Decentralization is mainly a transfer of authority, a handing down of responsibility from higher levels to more peripheral levels, for example via processes of *deconcentration* (transfer of authority from central levels of the Ministry of Health to field offices at a variety of levels) and *delegation* (transfer of authority from the Ministry to organizations not directly under its control, i.e. non-governmental agencies and citizens) [59,61]. Localization, however, puts greater emphasis on a third process associated with decentralization: *devolution*, or the creation and strengthening of local authorities, which are substantially independent with respect to a defined set of functions [59,61].

### Challenges of decentralization and lessons for the future

A limited body of research has evaluated the impact of decentralization on health systems, highlighting strengths and weaknesses that provide valuable lessons for health systems moving toward localization. Decentralization efforts launched after Alma-Ata have had mixed results, frequently not improving local service delivery or the standard of care in rural areas [34,35]. A number of challenges have emerged in the decentralization of health systems in LMICs, including 1) problems of capacity at lower levels of the system, 2) the reproduction of inequities within the health sector, and 3) difficulties in defining “decision space” and precisely allocating authority to different levels of the system.

First, there is a general consensus in the literature that *limited local capacity* can strongly undermine the desired outcomes of health sector decentralization. In a study of decentralization of health services in 17 districts in Pakistan, Bossert and Mitchell found that institutional and personal capacities were crucial to implementation success [56]. Capacities needed for service provision, including clinical, scientific, and administrative expertise, are likely to be rare at peripheral levels of the health system. Indeed, Prud’homme (1995) suggests that sectors with a high degree of “technicity”— managerial and expertise requirements — are among the most difficult to decentralize, the health sector certainly fitting into this category [61]. Furthermore, decentralization can potentially erode what capacities do exist at the local level, known as the “population base” problem: if hospitals or clinics do not serve a certain minimum population, providers’ skills in performing less-common procedures may deteriorate due to lack of use, with a negative impact on the quality of services [62].

Problems of capacity are likely to remain an important concern under localization: the weak capacity of local actors will pose a significant obstacle to identifying problems, designing and evaluating solutions, and implementing policies at the local level. Proactive efforts will be needed to build capacity in many rural settings in LMICs. Some functions of the health system may be more easily managed by lower-capacity local authorities than others: Bossert et al. found that health systems in Ghana and Guatemala underperformed when local authorities controlled technical aspects of logistics systems (e.g. information systems) but had improved performance when they were given more control over planning and budgeting [63]. Regarding medical expertise, heath systems will need to significantly expand “training of trainers” at the most peripheral levels to transmit clinical knowledge to CHWs and other local service providers. For administrative and logistical know-how, it may be useful to coordinate with other sectors, many of which may also be undergoing processes of localization as they face the same resource constraints. Such inter-sectoral collaboration is another long-standing principle of public health dating back to Alma-Ata [64]. For health systems, ministries of education and public administration will be important partners in building capacities amongst health personnel and the general public, for example in basic management and administrative techniques.

Second, decentralization policies have been criticized for *exacerbating inequities* in health care even though, as with localization, the primary goal is to improve access to and quality of health services for the rural and urban poor. By promoting self-financing mechanisms, decentralization policies inherently eliminate redistributive modalities within the health system, leaving regional disparities common to many LMICs unchecked [65]. Thus, in poorer areas with lower tax receipts, health systems may fail the test of “congruence;” that is, the local fiscal base may be insufficient to fund the activities assigned by the central level [63,64]. Decentralizing financial responsibility for health services through the use of user fees has been found to be highly regressive [54,66,67], and many countries have moved away from user fees in recent years, subsequently experiencing large increases in service uptake and health outcomes [63,64,67]. Decentralization policies can also promote inequity via political mechanisms, as when local elites take leadership roles, potentially leading to the hijacking of resources if transparency and accountability are not enforced [54,56,65].

Many of these concerns about replicating or exacerbating inequalities apply to localization as well. In the environmental sustainability literature, several authors warn of the “local trap,” the common assumption that local solutions are somehow better or “more just.” However, as Born and Purcell note, “localizing production simply empowers one set of actors (local ones) rather than other actors at higher scales” [68,69]. The experience of decentralization has shown that benefits often accrue to elites in settings with highly unequal power structures; therefore, moves toward localization must be accompanied by careful measures to ensure that resources are distributed equitably [70]. Such measures might include village governing boards or oversight committees, and indeed the literature on decentralization supports “stronger” forms of local control such as devolution of services to locally elected governments [56]. Transparency of proceedings is essential and could take the form of community meetings or written documentation, depending on context, but it must be mandated by the health hierarchy.

Lastly, the question of “*decision space*,” or the exact allocation of authority within health organizations, has complicated efforts at decentralization and will likely pose similar problems for localization [58]. Bossert defines decision space as “a complex determination of how much choice over different functions and use of funding local officials are allowed/provided from above … as well as power actually exercised in practice” [56]. Decentralization policies have often failed to precisely delineate responsibilities in ways that satisfy both these needs; according to Bossert, too much attention has focused on *who* is granted power rather than *what* exactly these powers consist of [56].

Managers in localized health systems must find ways to clearly define responsibility and decision-making autonomy at all levels, from local communities to the central administration, and for these different levels to productively interact. The study of decentralized logistics systems in Guatemala and Ghana mentioned above, identifying which authorities are most effectively allocated to the local level, is an example of the kind of work needed as health systems move toward localization [65]. Hess et al. have proposed “adaptive management” as a framework for the health system’s response to rising petroleum prices in the United States. Originating in the natural resources field, adaptive management is “useful for managing dynamic systems whose complexity complicates linear management decisions” [57]. Such management systems emphasize ongoing learning and continuous stakeholder input, with regular revision of management objectives, a range of management choices, and ongoing monitoring and evaluation [71]. In LMICs as well, these processes could provide benefits in the form of their inherent flexibility, allowing for health systems to make use of local resources and cater to local needs.

## Conclusion

The current dialogue on health systems’ strengthening is insufficient to meet the most pressing threats facing service delivery in LMICs – notably external threats such as global climate change and rising energy prices as conventional petroleum reserves are depleted. The health sector must acknowledge the complexity of these challenges and bring sufficient expertise to bear. Much more research is necessary to identify appropriate processes, frameworks, tools, and best practices, particularly since the experience of decentralization proves solutions to be highly context-dependent. Funding is becoming available for climate change adaptation and mitigation research and activities, for example under the Green Climate Fund. Localization of health systems would likely fall under the purview of such funding, aimed at moving toward low-emission and climate-resilient development [72].

Using the concept of localization, we propose a preliminary model for adapting to higher petroleum prices, while also mitigating climate change, by reducing health systems’ reliance on fossil fuels through a variety of mechanisms. The decline of conventional petroleum reserves will present manifold challenges for already fragile health systems; however, it also represents an opportunity to empower local communities in the production of their own health. Localization embodies many of the unrealized tenets of Alma-Ata, including local empowerment, multi-sectoral collaboration, and a focus on disadvantaged populations – as well as a way to move towards these ideals in an environmentally sustainable manner.

## Abbreviations

CHWs: Community health workers; IEA: International Energy Agency; LMICs: Low and middle income countries; LPG: Liquefied petroleum gas.

## Competing interests

The authors declare that they have no competing interests.

## Authors’ contributions

MP and PW carried out the initial literature review and wrote early drafts on the impact of rising energy prices on health systems in LMICs and the concept of localization. SD prepared the manuscript and carried out revisions with MP and PW. All authors read and approved the final manuscript.
